# A Longitudinal Evaluation of a Multimodal POCUS Curriculum in Pediatric Residents

**DOI:** 10.24908/pocus.v8i1.16209

**Published:** 2023-04-26

**Authors:** Reshma Sabnani, Celia S Willard, Carolina Vega, Zachary W Binder

**Affiliations:** 1 Department of Pediatric Emergency Medicine, UMass Chan Medical School Worcester, MA USA; 2 Department of Pediatric Emergency Medicine, Boston University Chobanian and Avedisian School of Medicine Boston, MA USA

**Keywords:** Pediatrics, POCUS, ultrasound curriculum, paediatric POCUS

## Abstract

**Introduction:** Pediatric residency programs often do not include a point of care ultrasound (POCUS) curriculum. We analyzed a novel POCUS curriculum for pediatric residents that incorporated an online question bank (QB), in addition to a traditional teaching model of didactic instruction and hands-on learning experience. **Methods:** Four high-yield POCUS topics were chosen: Focused Assessment by Sonography for Trauma (FAST), soft tissue, lung, and cardiac**. **Residents completed online multiple-choice quizzes before and after each of four in-person learning sessions, taught by ultrasound faculty and fellows. At the end of the academic year participants completed a knowledge retention quiz. Confidence surveys were administered to participants throughout the course of the study. Differences in means were compared by Student’s t-test. **Results:** Learners demonstrated post-intervention score improvement for each of the four modules. Retention testing demonstrated retained improvement for the soft tissue and cardiac modules, but not for the FAST module. Self-reported confidence increased across all four topics. **Conclusion: **A multimodal POCUS curriculum utilizing a combination of an online QB and in-person teaching demonstrated lasting knowledge for pediatric trainees.

## Background

Point of care ultrasound (POCUS) has become an integral part of Emergency Medicine clinical care 1, 2, 3, 4, 5. In addition, national POCUS standards have been developed for Emergency Medicine residency curricula, clinical credentialing and imaging criteria 6, 7, 8, 9, 10, 11, 12, 13. There has been significant discussion of the potential diagnostic and procedural benefits of incorporating POCUS in various areas of pediatrics including: General Pediatrics, Hospital Medicine, Intensive Care, Neonatology, Emergency Medicine 14, 15, 16. Topics with high-yield potential in pediatrics include cardiac evaluation for function; pulmonary evaluation for pneumothorax and pneumonia; soft tissue evaluation for cellulitis and abscess; and the focused assessment of trauma (FAST) 14, 15, 17. Given a lack of established standards for the education of pediatric residents, piloted topics were selected by the Pediatric Emergency Medicine Ultrasound Director. In addition to its many diagnostic capabilities, POCUS is well tolerated, timely, and incurs no radiation exposure, all of which are important benefits in pediatrics.

There is a demonstrated demand for POCUS education to be included in pediatric residencies both from the perspective of program leadership as well as from pediatric trainees. One survey of pediatric residents at a free-standing children’s hospital found that 96% felt that POCUS was an important skill in pediatrics 18. In a separate survey, pediatrics residents overwhelmingly felt that POCUS training should be required during residency, yet only 15% of respondents had received such training 19. A national survey of pediatric residency associate program directors showed that a majority of respondents felt that POCUS should be taught to their trainees 20.

As of 2021, POCUS training is not referenced in either the American Board of Pediatrics content specifications or ACGME requirements for Pediatric Residency 21, 22, 23. To date, a standardized curriculum for pediatric trainees has not been established. It is worth noting that many graduating medical students are receiving some exposure to POCUS in medical school 24. The aim of this study is to evaluate the efficacy of a novel POCUS curriculum for pediatric trainees that combines an online question bank (QB) with a traditional teaching model of didactic instruction and hands-on instruction.

## Methods

Learners were a cohort of first year pediatrics and internal medicine/pediatrics (med/peds) residents at an urban tertiary care institution (Table 1). Four POCUS topics were chosen for study: FAST, Soft Tissue, Lung, and Cardiac.

**Table 1:** Characteristics of Participants.

**Participants**	**n=12, no. (%)**
**Gender **	
**Female**	9 (75%)
**Male**	3 (25%)
**Program **	
**Pediatrics**	8 (66%)
**Pediatrics/Internal Medicine**	4 (33%)

Quizzes from the Core Ultrasound question bank were used to evaluate participants' knowledge 25. The Core Ultrasound website provides web-based POCUS training and assessment 25. As part of this pilot project, a subscription to the Core Ultrasound question bank was purchased for all participants. Core Ultrasound quizzes are 25 questions each, and are formatted as a POCUS video clip, followed by a multiple-choice question. Once users select an answer, an annotated explanation of the correct and incorrect answers is provided. Each quiz contains a unique set of questions. Prior to each in-person educational session learners were asked to complete a Core Ultrasound question bank (QB) quiz in the content area that was to be taught during the in-person session. These results served as the pre-intervention assessment. 

**Figure 1:**
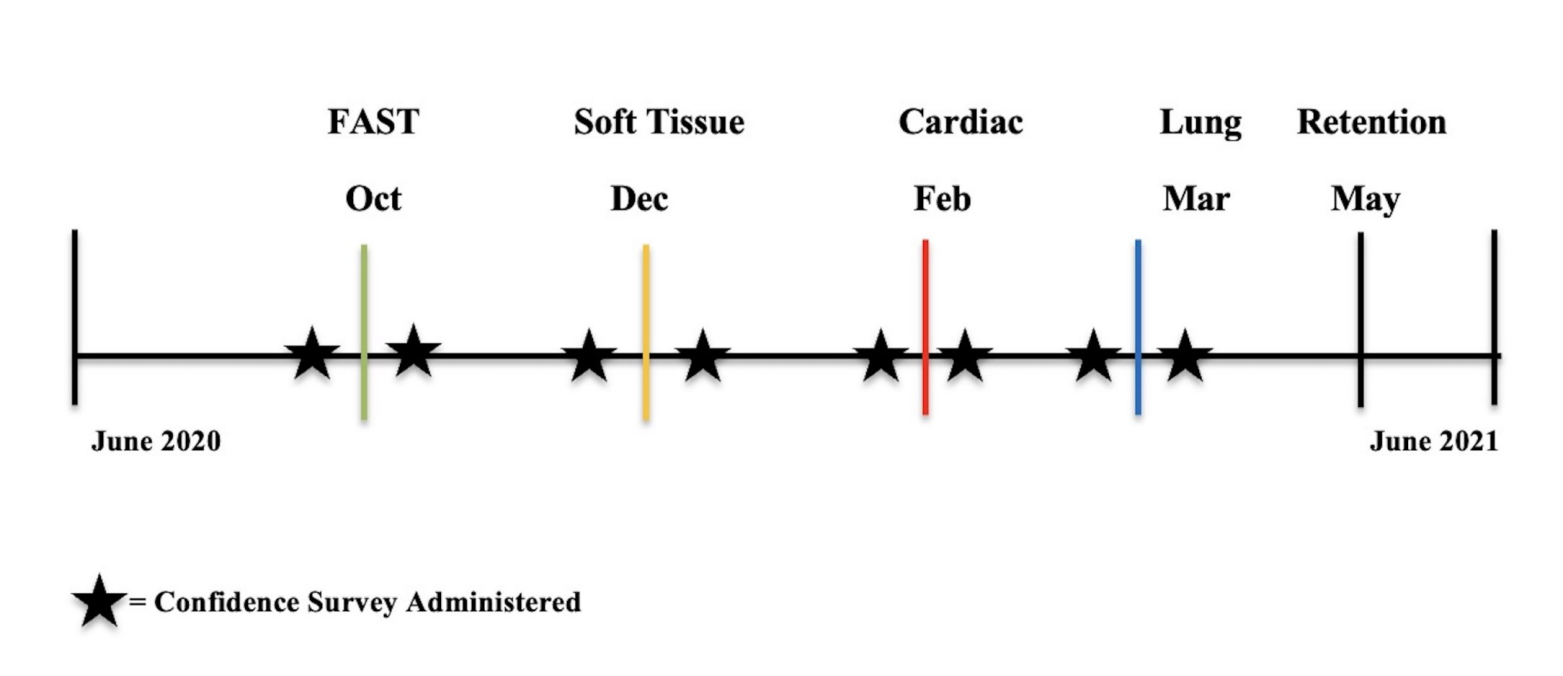
Study Timeline.

Following the pre-intervention assessment participants attended a 2-hour in-person educational session consisting of a didactic lecture followed by a hands-on skill session. The in-person educational sessions occurred throughout the 2021-2022 academic year: FAST in October, soft tissue in December, cardiac in February and lung in March (Figure 1). Each didactic lecture was 45 minutes and was provided by either the Pediatric Emergency Medicine Ultrasound director or Pediatric Emergency Ultrasound fellow. Each hands-on skill session, was performed on live models, and was led by the ultrasound faculty and fellows. During each skills session participants were able to practice the material that had just been taught during the didactic lecture, with directed feedback from the instructors. Immediately following the conclusion of each in-person educational session (didactic lecture and hands-on skill session), participants were asked to complete a second QB quiz, consisting of 25 unique questions, evaluating the content that had just been taught. These results served as a post-intervention assessment. The completion of the post-intervention quiz by participants served to both assess knowledge acquisition and consolidate learning.

At the end of the academic year participants were asked to complete three additional QB quizzes (FAST, soft tissue and cardiac). These quizzes consisted of 25 unique questions each (75 questions total). The results of these quizzes served as the retention assessment. Retention data was not collected for the lung module due to its proximity to the end of the academic year (Figure 1). 

Residents working nights, on vacation, or participating in away rotations were not able to attend all in-person educational sessions. All participants were asked to complete the pre-intervention quiz, post-intervention quiz, and retention quizzes. In addition to objectively measuring the participants progress via the quizzes, subjective assessment was made via self-reported confidence surveys. Participants' confidence in performing each of the four POCUS exams (FAST, cardiac, soft tissue, lung) was assessed using a Likert scale (0-11). Participants completed the confidence survey immediately before and immediately after each educational session resulting in eight data points per participant (Figure 1).

Statistical analysis was performed by the study’s senior author using Social Sciences Statistics (2018). Paired means were compared using Student’s t-test. The differences in paired means were determined to be normally distributed via The Kolmogorov-Smirnov Test of Normality.

We performed subgroup analysis of the FAST module to compare the results of participants who were able to attend the in-person educational session to those participants who were unable to attend the in-person session.

## Results

**Figure 2:**
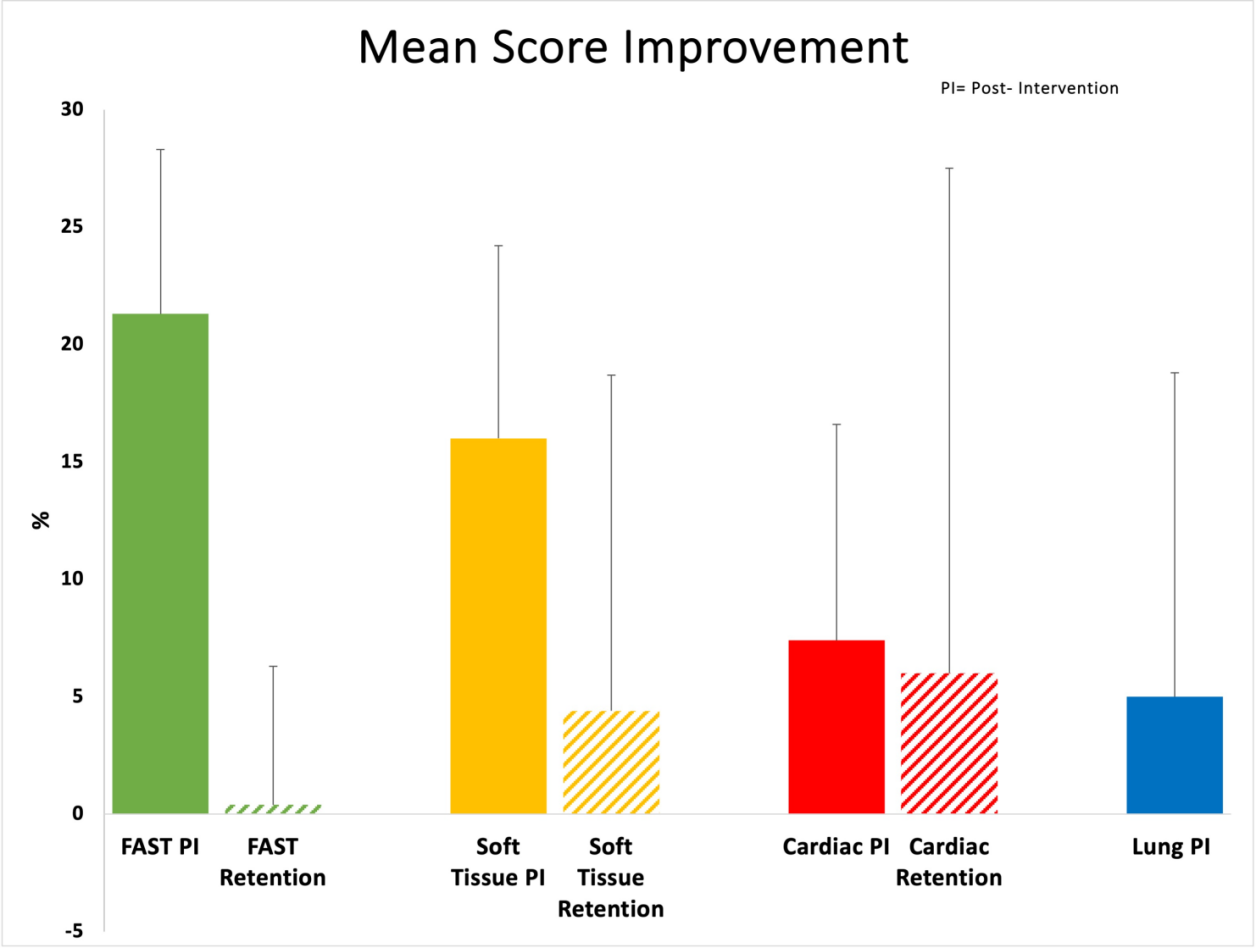
Mean Score Improvement for Post–Intervention and Retention Quizzes, including upper limit of 95% confidence interval.

There were twelve residents who participated in the curriculum. Learners demonstrated score improvement for each of the 4 POCUS modules. Participants on average improved their scores by an absolute value of 21.3% (95% CI 14.4% to 28.3%) for the FAST module, 16.0% (95% CI 7.8% to 24.2%) for the soft tissue module, 7.4% (-1.8% to 16.6%) for the cardiac module, and 5.0% (-8.8% to 18.2%) for the lung module (Table 2 and Figure 2). The mean score improvement for the FAST and soft tissue modules was statistically significant. Retention quizzes showed a mean score improvement, compared to pre-intervention quizzes, for the soft tissue module 4.4% (95% CI -9.9% to 18.7%), and cardiac module 6.0% (95% CI -15.5% to 27.5%) which lacked statistical significance. Retention quizzes for the FAST module did not show a mean score improvement compared to pre-intervention results: 0.4% (95% CI -5.4% to 6.3%) (Table 2 and Figure 2). 

**Table 2:** Mean Score Improvement for Post – Intervention and Retention Quizzes.

	**Post-Intervention Mean Improvement**	**95% CI**	**Retention Mean Improvement**	**95% CI**
**FAST**	21.3%*	14.4 to 28.3	0.4%	-5.4 to 6.3
**Soft Tissue**	16.0%*	7.8 to 24.2	4.4%	-9.9 to 18.7
**Cardiac**	7.4%	-1.8 to 16.6	6.0%	-15.54 to 27.54
**Lung**	5.0%	-8.8 to 18.2	NA	NA
* = statistically significant				

For the FAST module, 7 participants attended the in-person educational session, whereas 5 participants were unable to attend. Participants who attended the in-person educational session on average improved their post-intervention scores by an absolute value of 24.0% (95% CI 12.5% to 35.5%) compared to 17.6% (95% CI 7.3%-27.9%) in those who were unable to attend. The mean score improvement was statistically significant for both sub-groups (Table 3). 

**Table 3:** Subgroup Analysis of the FAST Module by In-Person Attendance.

	**Participants**	**Post-Intervention Mean Score Improvement**	**95% CI**
**FAST Attended In-Person**	7	24.0%*	12.5 to 35.5
**FAST Did Not Attend In-Person**	5	17.6%*	7.3 to 27.9

**Figure 3:**
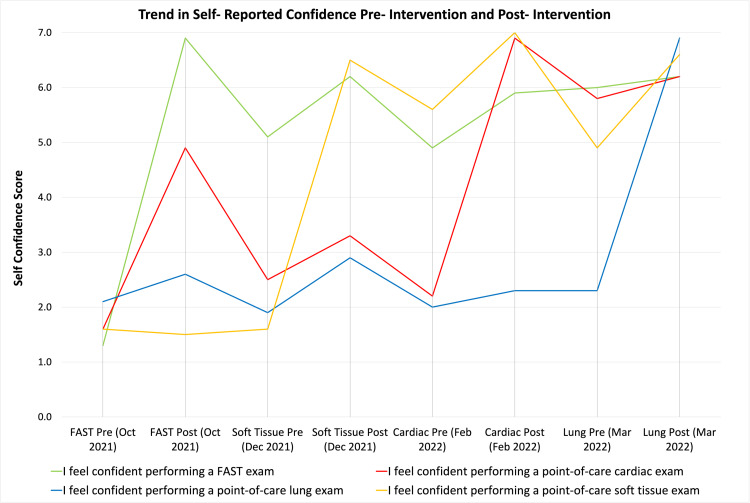
Trends in Self-Reported Confidence.

Figure 3 shows participant self-reported confidence trended over time. Increased participant confidence correlated with the teaching of that content (i.e. confidence in performing a FAST improved after the FAST module was taught). The increase in confidence for each module persisted through the end of the academic year. 

## Discussion

Participants showed mean score improvement for each of the four topics taught by our novel POCUS curriculum. The score improvement for the FAST and soft tissue modules showed statistical significance.

The greater mean score improvement in those participants that attended the FAST in-person educational session (24.0%) compared to those that did not attend (17.6%) suggests a benefit from the inclusion of in-person teaching modalities as opposed to the online QB alone (Table 3). 

Retention results demonstrated retained improvement for the soft tissue and cardiac modules, but not for the FAST module. We hypothesize that this is because the FAST session occurred earliest in the academic year, 7 months before the retention quiz, whereas the soft tissue and cardiac modules occurred 5 and 3 months prior to the retention quiz respectively (Figure 1). This suggests that more frequent interaction with the material, specifically through additional hands on sessions, is required for participants to retain learned content. In the future, retention quizzes could be administered at fixed intervals, for example 3 months after each module is taught, to compare retention results more validly across topics. Furthermore, the timing of the lung module, near the end of the academic year, did not allow for retention testing of that material. Given the clinical importance and applicability of lung POCUS in pediatrics, future iterations of this curriculum would likely benefit from moving the lung module to earlier in the academic year in order to ensure a complete evaluation of its content. 

Participants’ reported self-confidence increased after each hands-on session, and that confidence remained high for the remainder of the academic year (Figure 3). Interestingly, participant confidence in performing a FAST exam remained high through the end of the academic year despite the fact that participant multiple choice score improvement was not retained. This discrepancy may be due to the fact that participants were exposed to the FAST exam, in a clinical setting, while on Emergency Medicine rotations throughout the year, but were not re-exposed to the question bank content prior to the retention exam. This discrepancy supports the need for re-exposure to POCUS content throughout training.

A limitation of our study was that it was performed at a single institution. The study was further limited by the size of the first-year pediatric residency class (n=12). The residents were given protected time to attend in-person sessions, however residents working nights, on vacation, or participating in away rotations were not able to attend all in-person sessions.

Our curriculum was administered within a single academic year. A study by Brandt et al. 17 evaluated a POCUS curriculum delivered to first-year pediatric residents that utilized a traditional educational model of didactic instruction followed by hands on scanning. Participants in their study were assessed via pre and post testing and an objective structured clinical exam (OSCE) at the end of their first year of residency. Participants in their study were then required to complete 8 hours of content review and hands-on training in their 2^nd^ and 3^rd^ years of residency. The residents in their study rated this additional training as effective 3.9/4 (Likert scale). 

While our novel curriculum showed effectiveness, as evidenced by post- intervention score improvement, it would likely be enhanced by repeated exposure to learned content throughout training, so as to build retention. The declining scores on the FAST retention quizzes, which were assessed seven months after the corresponding in-person didactic session, supports this proposed improvement. 

This pilot study did not include a skills assessment. Multiple choice questions, as used in this study, can measure obtained knowledge. Given that POCUS is a practical skill, future study of this topic would benefit from the incorporation of a skill assessment, as was included by Brandt et al. 17 . Future studies and educational curricula may include second- and third-year residents, additional practice time for participants, the inclusion of a skills assessment and additional POCUS topics. 

## Conclusion

This study was able to demonstrate that our novel POCUS curriculum led to knowledge acquisition and improved confidence in pediatric trainees. There is a clear demand for POCUS education of pediatric residents, from both the perspective of program leadership and trainees, who recognize the importance of this skill and who desire POCUS training 19, 20, 21. We suggest that our novel curriculum combining QB and in-person learning could serve this demand.

## Statement of Ethics Approval

University of Massachusetts Medical School Institutional Review Board (IRB) approval was obtained.

## Disclosures

None.
